# Estimates of heritable and environmental components of familial breast cancer using family history information

**DOI:** 10.1038/sj.bjc.6603753

**Published:** 2007-05-01

**Authors:** E Couto, K Hemminki

**Affiliations:** 1 Department of Neurobiology, Care Sciences and Society, Karolinska Institute, CeFAM, Alfred Nobels Allé 12, Plan 5, 141 83 Huddinge, Sweden; 2 Division of Molecular Genetic Epidemiology, German Cancer Research Center (DKFZ), Im Neuenheimer Feld 580, 69120 Heidelberg, Germany

**Keywords:** familial breast cancer, heredity, environment, paternal/maternal lineage

## Abstract

Using the Swedish Family-Cancer Database, the increased risk of breast cancer in women with relatives with the disease did not vary with paternal/maternal lineage. Familial breast cancer heritable component was 73% and the environmental proportion 27%. Familial aggregation of breast cancer in women below age 51 years is mainly due to heritable causes.

Although having a mother or sister diagnosed with breast cancer is known to increase a woman's risk of the disease (Collaborative Group on Hormonal Factors in Breast Cancer, 2001), the mechanisms underlying this association remain unclear. A family represents a group of individual sharing a common environment and genes. Hence, the mechanisms leading to this higher risk of breast cancer could be environmental, genetic, or a combination of the two.

Twin and other family studies have consistently shown a familial aggregation of female breast cancer (Lichtenstein *et al*, 2000; Collaborative Group on Hormonal Factors in Breast Cancer, 2001; Hemminki and Granstrom, 2003; Amundadottir *et al*, 2004; Kerber and O'Brien, 2005; Baker *et al*, 2005). The familial risk of about 2.0 among first-degree relatives is so high that a substantial part of it must be caused by heritable factors (Hopper and Carlin, 1992; Lorenzo and Hemminki, 2005). One estimate suggests that heritable factors contribute to 27% of the total risk of breast cancer, and environmental factors to 73% (Lichtenstein *et al*, 2000). However, no corresponding studies have estimated these components in familial breast cancer. While, these proportions have mostly been measured using twin data, we use instead first- and second-degree familial relationships. As environmental sharing between second-degree relatives is probably low, the breast cancer risk associated with having affected second-degree relatives is assumed to be due to heritable causes. Breast cancers with a possible genetic cause have mainly been diagnosed at young ages (Hall *et al*, 1990; King *et al*, 1993), implying that the heritable component varies with age.

It is unknown whether breast cancer risk associated with family history differs according to the side of the family of the affected relative. Probably due to lack of reliable data, few studies have investigated this possible difference in risk (Macklin, 1959). However, such studies would clarify breast cancer inheritance pathways.

This population-based study investigated breast cancer risk among women having first- and second-degree relatives with the disease, in order to estimate the heritable and environmental components of familial breast cancer, according to women's age; risk was also examined according to paternal/maternal lineage.

## MATERIALS AND METHODS

The Swedish Family-Cancer Database was described in detail previously (Hemminki *et al*, 2001). This Database was created by linking information from the national multigeneration register, censuses, cancer registries and national deaths notification using the national 10-digit personal number. Histories of cancer among mothers, sisters, grandmothers and aunts were extracted from the multigeneration register. Information on both maternal and paternal grandmothers was available for 1 550 374 women; 868 of these, born between 1952 and 1979, had been diagnosed with invasive breast cancer (with age at diagnosis of 50 years or below). Each case was matched on year of birth and geographical region to four women free of invasive and *in situ* breast cancer and alive at the date of the case's diagnosis. Odds ratios (ORs) for invasive breast cancer associated with having an affected relative were estimated, using conditional logistic regression. Analyses were adjusted for mother's and father's year of birth, number of maternal and paternal aunts, and number of sisters. The analyses of familial breast cancer risk associated with second-degree family history according to paternal/maternal lineage were adjusted for the above variables excluding number of sisters. Differences between ORs were evaluated using standard *χ*^2^ heterogeneity tests.

These ORs were used to estimate heritable and environmental components of familial breast cancer. Owing to low environmental sharing among second-degree relatives, the risk associated with having affected second-degree relatives was assumed to be due to heritable causes. Hence, assuming a 50% gene sharing between first- and second-degree relatives, we concluded that the excess risk associated with first-degree family history should be equal to twice the excess risk associated with second-degree family history. This excess risk was considered as being due to heritable causes. If it was lower than the excess risk evaluated through conditional logistic regression, the difference was assumed to be due to environmental causes.

## RESULTS

**Figure 1:**
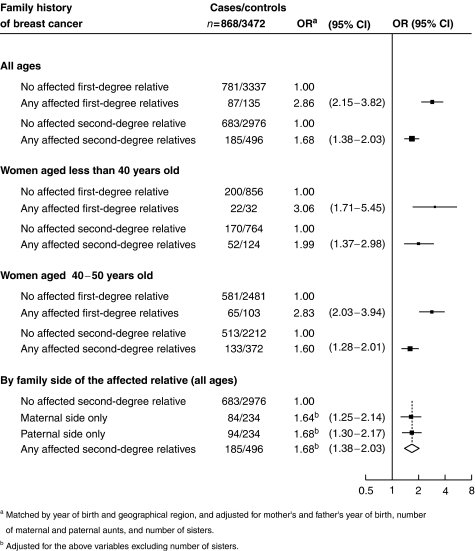
OR for breast cancer associated with family history of the disease.

Women with a first- and second-degree relative with breast cancer had a higher risk of contracting the disease than women without such a family history (Figure 1). This risk was higher when the affected relative is a mother and/or a sister(s) compared to a grandmother and/or an aunt(s). In women of all ages, the ORs for breast cancer associated with first- and second-degree family history were 2.86 (95% CI: 2.15–3.82) and 1.68 (1.38–2.03), respectively. Those risks were higher when women were of a younger age. Breast cancer risk did not vary with paternal/maternal lineage, the OR being 1.64 (1.25–2.14) and 1.68 (1.30–2.17) when the affected relative was on the mother's and father's side of the family, respectively (Figure 1).

Considering the breast cancer risk associated with having an affected second-degree relative as a correct estimate of familial breast cancer risk due to heritable causes, heritable and environmental components of breast cancer were estimated using the ORs presented in Figure 1 (see Table 1). Considering all women, familial breast cancers were 73% heritable and 26% environmental. The heritable component was 96 and 66% in women aged less than 40 and 40–50 years, respectively.

## DISCUSSION

Women with first- and second-degree relatives with breast cancer have a higher risk of the disease themselves. This risk is higher when the affected relative was a mother or a sister(s) compared to a grandmother or an aunt(s), as found in a meta-analysis of 74 studies (Pharoah *et al*, 1997). Family history of breast cancer was extracted from national registries and did not rely on reporting of affected relatives as in most studies. The quality of reporting was found to worsen with increasingly distant relatives (Ziogas and Anton-Culver, 2003), indicating possible unreliability of the data in such studies.

Few studies have investigated whether breast cancer risk varies according to paternal/maternal lineage (Macklin, 1959). In the present study, the risk of breast cancer associated with having affected second-degree relatives did not vary according to paternal/maternal lineage, which is consistent with the failure to identify sex-linked genes in breast cancer.

This report uses a novel method to estimate heritable and environmental components of a disease. Although these proportions have mostly been measured using twin data, we used first- and second-degree family history. Overall, familial breast cancer was found to be 73% heritable and 26% environmental, suggesting that familial aggregation in young breast cancers is mainly due to heritable factors. Previous studies have found similar results (Hopper and Carlin, 1992; Hemminki and Granstrom, 2003). The heritable component of familial breast cancers increased with decreasing women's age, with 96 and 66% of familial breast cancers in women aged below 40 and 40–50 years, respectively. Factors explaining breast cancer familial aggregation might differ according to age. Participants included in the present report were born between 1952 and 1979, with age at diagnosis ranging between 16 and 50 years old, and different estimates of heritable and environmental components of familial breast cancer might be observed in older cases.

The Swedish Family-Cancer Database includes information on most of the Swedish population, providing results, which are generalisable to a population of a similar age to the one discussed. The results presented here suggest that familial aggregation in young breast cancers is mainly due to heritable causes.

## Figures and Tables

**Table 1:** Heritable and environmental components of familial breast cancer according to the ORs for breast cancer associated with first- and second-degree FH by women's age

**OR for breast cancer associated with second-degree FH**	**Corresponding excess risk**	**Corresponding ORs for breast cancer associated with first-degree FH^a^**	**Observed ORs for breast cancer associated with first-degree FH**	**Heritable component (%)**^b^, ^c^	**Environmental component (%)** d
*All ages*					
1.68	0.68	1.36 (0.68 × 2)	1.86	1.36 **(73%)**	0.50 (1.86–1.36) **(27%)**
					
*Women aged less than 40 years*					
1.99	0.99	1.98 (0.99 × 2)	2.06	1.98 **(96%)**	0.08 (2.06–1.98) **(4%)**
					
*Women aged 40–50 years*					
1.60	0.60	1.20 (0.60 × 2)	1.83	1.20 **(66%)**	0.63 (1.83–1.20) **(34%)**
					

FH=family history; OR=odds ratio.

a These estimates were obtained using the excess risk corresponding to the ORs for breast cancer associated with second-degree family history.

b The heritable component is assumed to be equal to the third column.

c The percentages were obtained dividing the third by the fourth column.

d The percentage was obtained by substracting the percentage of the fifth column to 100.
